# Elevated Red Blood Cell Distribution Width as a Simple Prognostic Factor in Patients with Symptomatic Multiple Myeloma

**DOI:** 10.1155/2014/145619

**Published:** 2014-05-21

**Authors:** Hyewon Lee, Sun-Young Kong, Ji Yeon Sohn, Hyoeun Shim, Hye Sun Youn, Sangeun Lee, Hyun Ju Kim, Hyeon-Seok Eom

**Affiliations:** ^1^Hematologic-Oncology Clinic, Center for Specific Organs Cancer, Research Institute and Hospital, National Cancer Center, 323 Ilsan-ro, Ilsandong-gu, Goyang, Gyeonggi-do 410-769, Republic of Korea; ^2^Department of Laboratory Medicine, Center for Diagnostic Oncology, Research Institute and Hospital, National Cancer Center, 323 Ilsan-ro, Ilsandong-gu, Goyang, Gyeonggi-do 410-769, Republic of Korea

## Abstract

Red blood cell distribution width (RDW) is a parameter reported in complete blood cell count tests, and has been reported as an inflammatory biomarker. Multiple myeloma (MM) is known to be associated with inflammatory microenvironments. However, the importance of RDW has been seldom studied in MM. For this study, 146 symptomatic myeloma patients with available RDW at diagnosis were retrospectively reviewed, and their characteristics were compared between two groups, those with high (>14.5%) and normal (≤14.5%) RDW. RDW was correlated to hemoglobin, MM stage, **β**2-microglobulin, M-protein, bone marrow plasma cells, and cellularity (*P* < 0.001). During induction, overall response rates of the two groups were similar (*P* = 0.195); however, complete response rate was higher in the normal-RDW group than it was in the high-RDW group (*P* = 0.005). With a median follow-up of 47 months, the normal-RDW group showed better progression-free survival (PFS) (24.2 versus 17.0 months, *P* = 0.029) compared to the high-RDW group. Overall survival was not different according to the RDW level (*P* = 0.236). In multivariate analysis, elevated RDW at diagnosis was a poor prognostic factor for PFS (HR 3.21, 95% CI 1.24–8.32) after adjustment with other myeloma-related prognostic factors. RDW would be a simple and immediately available biomarker of symptomatic MM, reflecting the systemic inflammation.

## 1. Introduction

Red blood cell distribution width (RDW) is one of the parameters routinely reported in the complete blood cell count test, and it reflects the size variability of mature erythrocytes in peripheral blood and ineffective erythropoiesis of bone marrow [1]. It has been used in routine practice for several decades to make a differential diagnosis for various cases of anemia, such as an iron deficiency anemia [2, 3]. Recently, RDW has been reported as an inflammatory biomarker in various conditions such as cardiovascular diseases [4, 5], acute and chronic kidney diseases [6, 7], chronic pulmonary diseases [8], and critically ill patients [9–12]. In these conditions, elevated RDW level could predict severe morbidity and mortality. Furthermore, RDW could reflect subclinical inflammation and it is associated with poor functional status dependence in the elderly [13].

Multiple myeloma is the hematologic malignancy originating from plasma cells; it is characterized by increased monoclonal protein (M-protein) and specific organ injuries resulting in hypercalcemia, anemia, renal insufficiency, and osteolytic bone lesions. The median age at diagnosis of multiple myeloma is greater than 65 years, and its incidence rapidly increases by age. The prognostic factors associated with multiple myeloma mainly reflect plasma cell burden or intrinsic characteristics of the myeloma clones. The International Staging System (ISS) and cytogenetic risk groups are well known as important prognostic models [14–16].

Inflammatory parameters such as C-reactive protein (CRP) and interleukin-6 (IL-6) at diagnosis have been also reported as prognostic in patients with multiple myeloma [17, 18]. Multiple myeloma is one of the malignancies that are associated with inflammatory microenvironments [19, 20]. Novel therapies targeting inflammatory cytokines and tumor microenvironment have been investigated in patients with multiple myeloma [21, 22]. Acute kidney injury induced by the free light chains in multiple myeloma is also associated with a cascade of inflammatory responses [23].

With regard to these characteristics of multiple myeloma, we hypothesized that RDW has a prognostic value in patients with multiple myeloma. We expected that RDW would reflect not only the tumor burden but also the global condition of the patients, including comorbidities such as age, risk of cardiovascular complications, and severity of renal impairment. Use of RDW in patients with multiple myeloma has seldom been studied; therefore, we performed a retrospective review to investigate the prognostic value of baseline RDW level at diagnosis in patients with symptomatic multiple myeloma.

## 2. Materials and Methods

### 2.1. Patients

This analysis included patients with multiple myeloma who were diagnosed and treated at the National Cancer Center, Goyang, Korea, between 2005 and 2012. Patients who were older than 20 years with previously untreated symptomatic multiple myeloma, who had been administered at least one dose of systemic chemotherapy and who had complete blood cell test results available and a reported RDW level before treatment, were enrolled. Medical records and laboratory results were retrospectively reviewed.

The diagnosis of symptomatic multiple myeloma was made when the patient had (a) 10% or more clonal plasma cells on bone marrow examination or a biopsy-proven plasmacytoma, (b) serum and/or urinary monoclonal protein (except in nonsecretory patients), and (c) evidence of end-organ damage that is related to multiple myeloma [24]. Stage was classified by the ISS [14], and a response assessment was performed based on the criteria from the International Myeloma Working Group [24]. Patients with hypodiploidy or −13 by conventional chromosome analysis were regarded as high risk. Cytogenetic abnormalities detected by fluorescent* in situ* hybridization (FISH) such as t(4;14), t(14;16), or del(17p) were also designated as high risk [24]. Data, including patients' demographics, known prognostic factors for multiple myeloma, treatments, and clinical outcomes, were collected with RDW level at the time of the first systemic chemotherapy. This study was approved by the institutional review board of the National Cancer Center, Korea, and conducted according to the Declaration of Helsinki.

### 2.2. Measurement of RDW

Baseline RDW level at diagnosis was defined as the value that was obtained on the nearest day within 2 weeks before the front-line treatment. RDW was measured using XE-2100 (Sysmex, Kobe, Japan). RDW is reported as a coefficient of variation (percentage) of red blood cell volume. The reference range for RDW in our institution is 11.5% to 14.5%. We defined that the RDW level was “high” when it was >14.5%.

### 2.3. Statistical Analysis

Based on pretreatment RDW levels, patients were divided into the high-RDW group (>14.5%) and normal-RDW group (≤14.5%). Between the two groups, patients' characteristics and survival outcomes were compared. Continuous and categorical parameters were analyzed using independent sample* t*-tests and *x* ^2^ tests, respectively. For survival analysis, the Kaplan-Meier method with a log-rank test was used. Progression-free survival (PFS) was measured from the date of the first treatment to the earliest date that the progression of multiple myeloma or death was documented. Initially planned induction therapy, high dose chemotherapy with autologous stem cell transplantation, and maintenance therapy were regarded as the front-line therapy. Stem cell transplantation was not censored in this survival analysis. Overall survival (OS) was defined as the duration from the first treatment to all-cause death. The prognostic value of pretreatment RDW level was validated using the Cox proportional hazards model. The significant variables with *P* < 0.05 defined in univariate survival analyses (by log-rank test) and previously well-known prognostic factors in patients with multiple myeloma such as age, performance status, stage at diagnosis, cytogenetic risk group, type of induction therapy, and stem cell transplantation were included for the multivariate analysis to validate the prognostic value of RDW. Differences were considered statistically significant when two-sided* P* values were <0.05.

## 3. Results

### 3.1. Patient Characteristics

A total of 146 patients were eligible for this analysis. The median age was 61 (32–83) years, and 91 (62.3%) were male. The mean baseline RDW level was 14.6%, and it ranged from 11.9% to 22.0%. Among these, 55 (27.7%) patients presented an RDW higher than the upper limit of normal range (>14.5%). The mean RDW values of normal-RDW group and high-RDW group were 13.3% (range, 11.9–14.5%) and 16.8% (range, 14.6–22.0%), respectively. Characteristics of the patients stratified according to the pretreatment RDW level are presented in Table 1. High-RDW group included more elderly patients compared to normal-RDW group, although it was not statistically significant (*P* = 0.061). The distribution of comorbidities such as diabetes mellitus, hypertension, cardiovascular diseases, malignancies other than multiple myeloma, chronic liver disease, and chronic pulmonary diseases was not different between the two groups.

**Figure 1:**
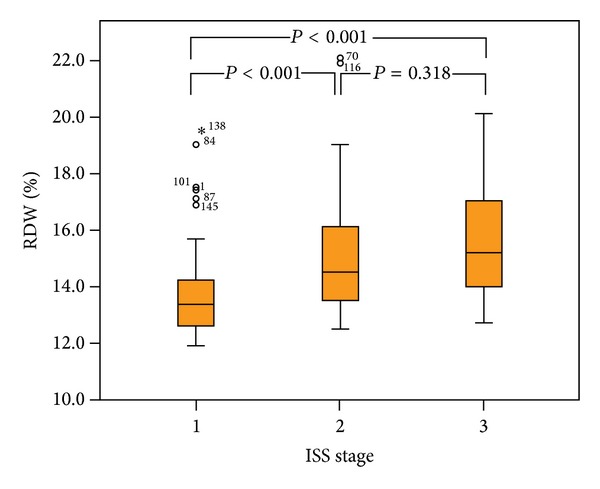
Red blood cell distribution width (RDW) level at diagnosis of multiple myeloma according to the International Staging System (ISS).

Baseline RDW level correlated to hemoglobin (negative correlation, *ρ* = −0.593, *P* < 0.001), albumin level (negative correlation, *ρ* = −0.386, *P* < 0.001), serum creatinine level (*ρ* = 0.208, *P* = 0.016), *β*2-microglobulin (*ρ* = 0.443, *P* < 0.001), M-protein level (*ρ* = 0.289, *P* = 0.002), bone marrow plasma cell burden (*ρ* = 0.370, *P* < 0.001), and bone marrow cellularity (*ρ* = 0.262, *P* = 0.002). Patients with ISS-I disease presented with lower RDW (mean ± SD, 13.75% ± 1.69) compared to ISS-II (mean ± SD, 15.05% ± 2.19, *P* < 0.001) and ISS-III (mean ± SD, 15.61% ± 2.11, *P* < 0.001) patients (Figure 1). Extramedullary plasmacytoma was more frequent in the normal-RDW group compared to high-RDW group (44.0% versus 20.0%, *P* = 0.004).

Cytogenetic data based on conventional chromosome analysis and FISH were available for 108 (74.0%) patients. Twenty-one (19.4%) of them were stratified as high risk. The proportion of high-risk patients in the normal-RDW and high-RDW groups was not statistically different (17.4% versus 23.1%, *P* = 0.613).

The front-line treatment for symptomatic myeloma is shown in Table 1. Five (3.4%) patients received radiation therapy without any systemic chemotherapy. Ninety patients (61.6%) were administered with novel agents such as thalidomide, lenalidomide, and bortezomib as an induction regimen. Others (34.9%) received high-dose steroids alone or conventional chemotherapy, such as doxorubicin or vincristine. Among the evaluable patients, the overall response rates (ORR) were not different between the normal-RDW and high-RDW groups (82.9% versus 73.1%, *P* = 0.195). However, the complete response (CR) rate was significantly higher in the normal-RDW group compared to the high-RDW group (36.6% versus 13.5%, *P* = 0.005). After induction, autologous stem cell transplantation was performed in 43 (29.5%) patients. Among them, 31 (34.1%) were in the normal-RDW group and 12 (21.8%) were in the high-RDW group (*P* = 0.136).

### 3.2. Association between RDW Level and Clinical Outcomes

**Figure 2:**
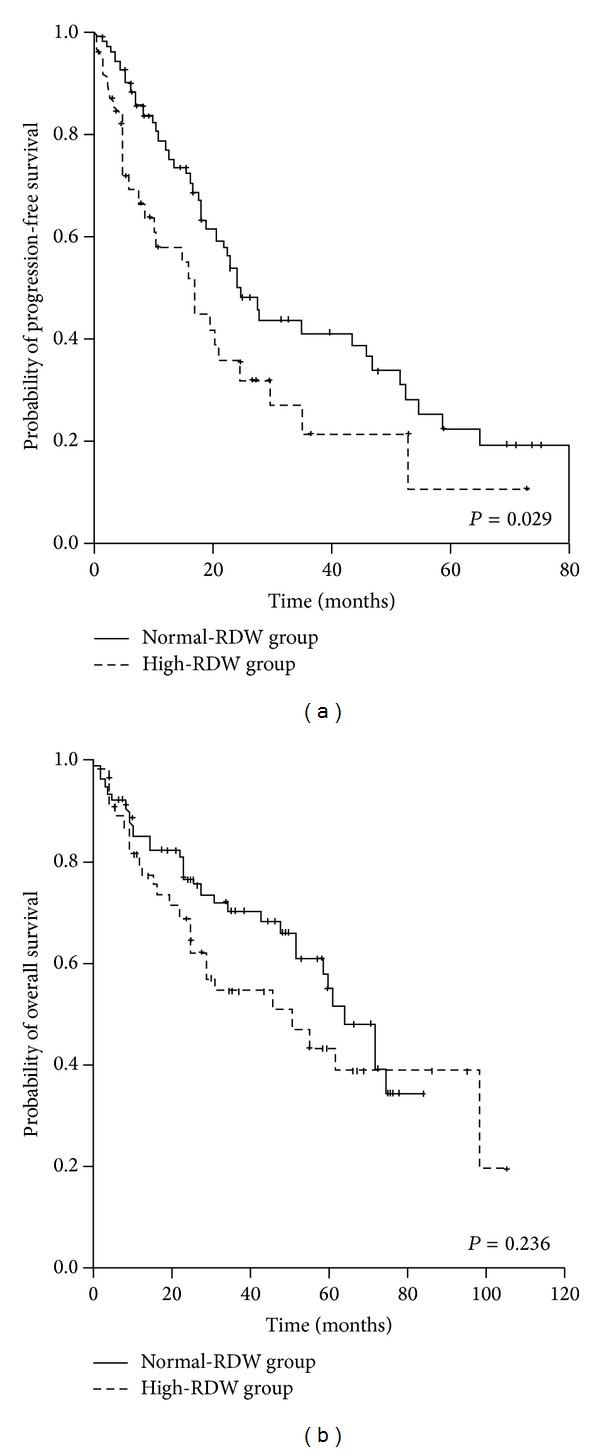
Survival curves according to red blood cell distribution width level at diagnosis in patients with symptomatic multiple myeloma.

With a median follow-up of 47 (3–104) months, patients with normal-RDW showed better progression-free survival compared to high-RDW patients (median PFS, 24.2 versus 17.0 months, *P* = 0.029). Overall survival showed a similar tendency between the two groups; however, the difference was not statistically significant (median OS, 63.6 versus 50.6 months, *P* = 0.236) (Figure 2).

Univariate analyses were performed to investigate the prognostic factors affecting disease progression and death (Table 2). Baseline RDW level (HR 1.69, 95% CI 1.05–2.75, *P* = 0.031), performance status (HR 1.89, 95% CI 1.05–3.41, *P* = 0.034), hemoglobin level (HR 0.88, 95% CI 0.79–0.99), albumin level (HR 0.56, 95% CI 0.36–0.86, *P* = 0.008), lactate dehydrogenase (LDH) level (HR 1.84, 95% CI 1.00–3.38, *P* = 0.050), and *β*2-microglobulin level (HR 1.08, 95% CI 1.03–1.14, *P* = 0.002) were potential risk factors for poor progression-free survival. RDW was not prognostic for overall survival (*P* = 0.238). Other potential prognostic factors for overall survival in this analysis are shown in Table 2.

To exclude the effect of anemia on RDW level, we performed a subgroup analysis according to the hemoglobin level. Patients with hemoglobin >10.0 g/L and RDW >14.5% showed worse outcomes (*P* = 0.024 for PFS, *P* = 0.121 for OS) compared to patients with hemoglobin >10.0 g/L and RDW ≤14.5%. These trends were not observed in patients with hemoglobin ≤10.0 g/L (*P* = 0.394 for PFS, *P* = 0.652 for OS).

**Figure 3:**
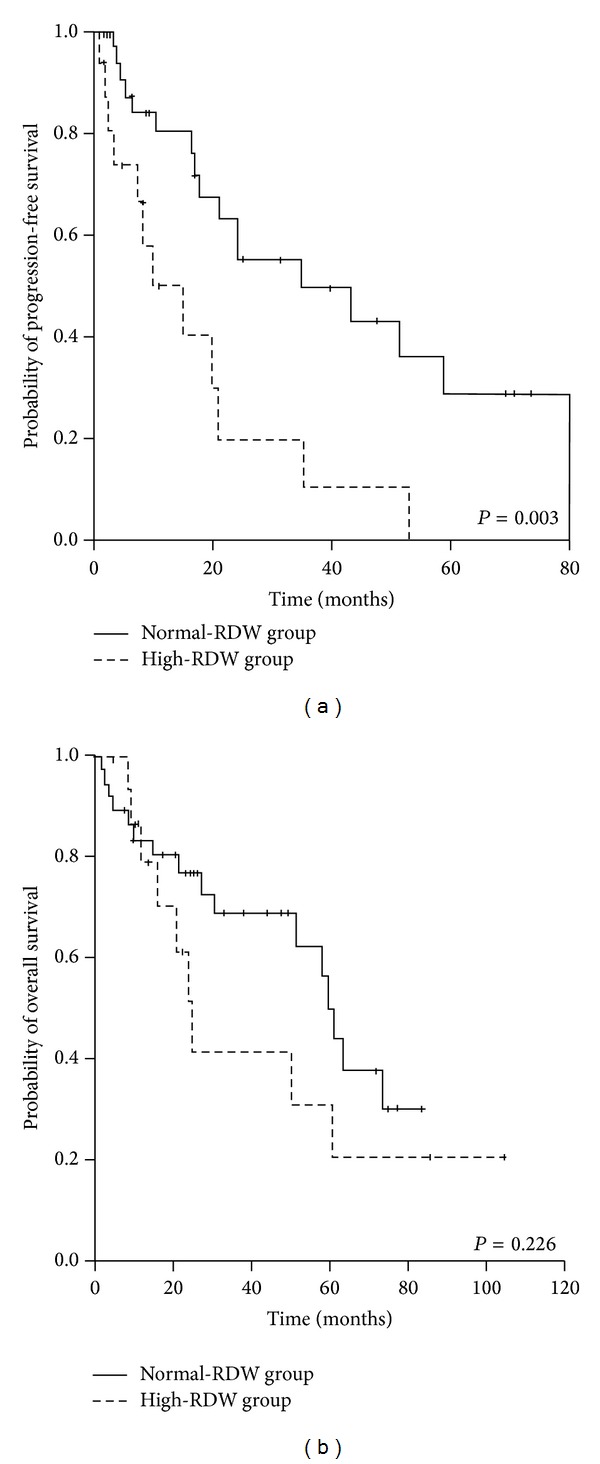
Survival curves according to red blood cell distribution width level at diagnosis in patients with symptomatic multiple myeloma treated with thalidomide-based induction.

We also performed a subgroup analysis with 53 cases who were transplant-eligible and who received thalidomide-based induction to validate the prognostic value of baseline RDW level in a homogeneous population. In this subgroup analysis, normal-RDW patients were associated with prolonged PFS compared to high-RDW patients (median PFS, 34.7 versus 10.2 months, *P* = 0.003); however, they did not show significantly better overall survival (60.5 versus 25.0 months, *P* = 0.266) (Figure 3).

RDW at diagnosis in patients with symptomatic multiple myeloma was found to be an independent predictor for disease progression or death by multivariable analysis (Table 3). Patients who had RDW >14.5% at diagnosis were associated with higher risk of disease progression or death with a hazard ratio (HR) of 3.04 (95% CI 1.16–8.01, *P* = 0.024) compared to patients with normal RDW at diagnosis. The other factors that revealed independent predictors of progression-free survival in this analysis set were cytogenetic risk group (high risk, HR 3.78, 95% CI 1.50–9.56, *P* = 0.005) and type of induction regimen (novel agents, HR 0.37, 95% CI 0.16–0.86, *P* = 0.020).

In multivariate analysis for overall survival, RDW at diagnosis was not an independent prognostic factor (HR 0.90, 95% CI 0.36–2.26) after adjustment with age, performance status, cytogenetic risk group, ISS, LDH, hemoglobin, albumin, *β*2-microglobulin, type of treatment, and autologous stem cell transplantation. As a result, cytogenetic risk group (high risk, HR 4.24, 95% CI 1.12–16.09), *β*2-microglobulin (HR 1.14, 95% CI 1.04–1.26), type of induction regimen (novel agents, HR 0.21, 95% CI 0.07–0.60), and autologous stem cell transplantation (performed, HR 0.05, 95% CI 0.01–0.52) were significantly associated with overall survival.

## 4. Discussion

The present study showed that RDW level at diagnosis was associated with poor prognosis in patients with symptomatic multiple myeloma. As far as we are aware, this study is the first report to evaluate the prognostic value of RDW in patients with multiple myeloma. We showed that the patients whose RDW level was high at diagnosis experienced shorter progression-free survival compared to patients with relatively low RDW. Progression-free survival is an important surrogate marker of long-term survival in patients with multiple myeloma. Although it was not statistically significant in the presented data, overall survival in the high-RDW group seemed to be shorter compared to the normal-RDW group. Analysis for overall survival is complicated because there may be more confounding factors influencing on clinical outcomes during the long follow-up duration.

In patients with multiple myeloma, RDW level might be influenced by anemia. Anemia is one of the major symptoms of multiple myeloma together with hypercalcemia, renal insufficiency, and osteolytic bone lesions, also called CRAB signs. However, we showed that RDW was well correlated not only to the hemoglobin level (negative correlation) but also to other parameters for high tumor burden such as azotemia, M-protein, bone marrow plasma cell percentages, and ISS stages. Furthermore, anemia of multiple myeloma does not simply reflect a decrease in red cell counts, but it is also associated with impaired iron release from reticuloendothelial macrophages, which can be observed in anemia of inflammatory conditions [25]. This suggests that RDW can reflect the overall inflammatory condition of multiple myeloma, partly influenced by combined anemia.

In line with this, there is an interesting report suggesting that hematological and inflammatory parameters, including RDW, can discriminate patients with cancer from patients without cancer in involuntary weight loss [26].

It is not surprising that RDW is prognostic in patients with multiple myeloma when we consider that it can reflect tumor burden and inflammatory conditions. We found that RDW at diagnosis was an independent prognostic factor for disease progression or death, even after the adjustment with other myeloma-associated parameters. An assessment of RDW level to predict clinical outcomes in patients with symptomatic myeloma has advantages. It can be acquired immediately when the patient is suspected of multiple myeloma to assess the patient's general condition objectively in the context of various comorbidities such as age, acute kidney injury, cardiovascular diseases, infectious condition, and malnutrition. RDW is significantly associated with increased risk in patients with heart failure [27, 28], kidney injury [6], and venous thromboembolism [29, 30], which are often encountered in myeloma patients.

Despite the increasing evidence for RDW as a prognostic factor in patients with inflammatory conditions, there are few reports addressing it in the area of oncology. Recently, there have been a few articles about the significance of RDW as a cancer biomarker. Although the prognostic value of RDW level on specific cancer types has not been studied well, there have been some reports on breast cancer and lung cancer. Seretis et al. showed that RDW was significantly higher in patients with invasive breast cancer compared to the patient with fibroadenomas. Elevated RDW showed remarkable correlation with the size of primary tumor, the number of axillary lymph nodes, and HER2 overexpression [31]. Warwick et al. showed that preoperative RDW in patients undergoing pulmonary resections for non-small-cell lung cancer could predict mortality and long-term survival [32]. In addition, Koma et al. showed that high RDW level was associated with poor survival in patients with lung cancer [33].

As shown in our data, it has been known that RDW increases with age [34]. Increased age can be a confounding factor which could mislead to conclude that RDW is prognostic. In our data, age itself was not associated with poor prognosis of myeloma, and RDW was a significant predictive biomarker for disease progression or death even after adjustment with other confounding factors including age in multivariate analysis. With regard to both patient's age and different antimyeloma treatment according to the age at diagnosis, which determines transplant-eligible or not, we also have described the results of subgroup analysis in transplant-eligible patients who received thalidomide-based induction in the paper.

There are several limitations in this analysis. First, there may be potential bias and inaccuracy in data collection, as in most retrospective analyses. Second, patient characteristics such as treatment regimens were heterogeneous. Third, we could not find the significant correlation between RDW and CRP, an important and commonly used inflammatory marker, in our dataset. Unfortunately, there were too many missing data because CRP level was not routinely checked at diagnosis. To validate the correlation between RDW and CRP, further prospective study is warranted. Also, we only focused on RDW level at diagnosis and did not evaluate the value of dynamic change in RDW level during the disease courses. A single measurement of RDW could not account for possible variation over time and could not predict overall survival, which may be influenced by various confounding factors. Finally, the value of RDW in prediction of poor prognosis may be slightly different according to the population, because presented RDW data were collected at a single center.

Despite the limitations, this is the first documentation on the prognostic value of RDW in patients with multiple myeloma with long-term follow-up. Further prospective analysis with mechanism studies is necessary to use it widely as a practical biomarker of multiple myeloma.

## 5. Conclusion

Elevated RDW at diagnosis in patients with symptomatic multiple myeloma was associated with advanced disease status and poor prognosis. It would be a novel and immediately available biomarker of the activity of multiple myeloma. Although we do not know the precise mechanism, it may reflect both the inflammatory status of myeloma itself and the patient's general condition. This easy and cost-effective biomarker may be useful particularly in practice.

## Figures and Tables

**Table 1:** Clinical characteristics of multiple myeloma patients.

	Total (*n* = 146)	Normal-RDW (*n* = 91)	High-RDW (*n* = 55)	*P*
RDW level, mean (range)	14.6 (11.9–22.0)	13.3 (11.9–14.5)	16.8 (14.6–22.0)	<0.001
Age, mean (range)	61 (32–83)	60 (32–83)	63 (41–80)	0.061
Sex, male/female	91/55	59/32	32/23	0.482
ECOG (≥2)	26/144 (18.1%)	14/80 (17.5%)	42/12 (22.2%)	0.373
Comorbidity				
Diabetes mellitus	13 (8.9%)	7 (8.0%)	6 (10.0%)	0.771
Hypertension	39 (26.7%)	24 (27.6%)	15 (25.0%)	0.850
Cardiovascular diseases	6 (4.1%)	4 (4.6%)	2 (3.3%)	1.000
Malignancies	12 (8.2%)	9 (10.3%)	3 (5.0%)	0.361
Chronic liver diseases	5 (3.4%)	3 (3.4%)	2 (3.3%)	1.000
Chronic lung diseases	6 (4.1%)	2 (2.3%)	4 (6.8%)	0.226
Hemoglobin, g/dL	10.7 (5.3–16.4)	11.4 (6.2–16.4)	9.5 (5.3–14.4)	<0.001
Platelet, ×10^9^/L	218 (37–691)	224 (68–555)	210 (37–691)	0.410
Creatinine, mg/dL	1.6 (1.0–9.0)	1.4 (1.0–7.0)	1.8 (1.0–9.0)	0.083
Calcium, mg/dL	9.0 (6.8–13.7)	9.2 (6.8–13.7)	8.8 (7.2–11.1)	0.004
Albumin, g/dL	3.8 (2.3–4.9)	3.9 (2.5–4.9)	3.5 (2.3–4.7)	<0.001
LDH, IU/L	199 (54–1832)	203 (54–1832)	192 (77–587)	0.762
B2MG, mg/dL	5.0 (1.2–41.9)	3.8 (1.2–18.7)	7.2 (1.6–41.9)	<0.001
CRP, mg/dL	1.20 (0.01–8.65)	0.99 (0.01–5.45)	1.53 (0.01–8.65)	0.204
ISS				<0.001
I	60 (41.7%)	50 (55.6%)	10 (18.5%)	
II	49 (34.0%)	26 (28.9%)	23 (42.6%)	
III	35 (24.3%)	14 (15.6%)	21 (38.9%)	
Unknown	2	1	1	
M-protein, g/dL	2.47 (0.01–9.31)	2.06 (0.08–6.80)	3.12 (0.01–9.31)	0.006
Light chain disease	31 (21.2%)	20 (22.0%)	11 (20%)	1.000
Nonsecretory type	5 (3.4%)	5 (5.5%)	0	0.157
Plasmacytoma	51 (34.9%)	40 (44.0%)	11 (20.0%)	0.004
Cytogenetic risk (high)	21/108 (19.4%)	12/69 (17.4%)	9/39 (23.1%)	0.613
Front-line treatment				0.606
Radiation only	5	2	3	
Thalidomide-based	63	44	19	
Bortezomib-based	14	9	5	
Bortezomib + thalidomide	9	4	5	
Lenalidomide-based	4	1	3	
Others	51	31	20	
ASCT	43 (29.5%)	31 (34.1%)	12 (21.8%)	0.136

RDW: red blood cell distribution width; ECOG: Eastern Cooperative Oncology Group; LDH: lactate dehydrogenase; B2MG: *β*2-microglobulin; ISS: International Staging System; ASCT: autologous stem cell transplantation.

**Table 2:** Univariate analyses for progression-free survival and overall survival.

	PFS			OS		
	HR	95% CI	*P*	HR	95% CI	*P*
RDW (%)	**1.69**	**1.05–2.75**	**0.031**	—	—	**0.238**
Age (year)	—	—	0.173	1.04	1.02–1.07	0.001
Sex (male)	—	—	0.591	—	—	0.835
ECOG (≥2)	1.89	1.05–3.41	0.034	1.82	1.01–3.28	0.048
Hemoglobin (g/dL)	0.88	0.79–0.99	0.028	0.86	0.76–0.99	0.029
Platelet (×10^9^/L)	—	—	0.633	0.99	0.99-1.00	0.001
Creatinine (mg/dL)	—	—	0.539	—	—	0.127
Calcium (mg/dL)	—	—	0.435	—	—	0.443
Albumin (g/dL)	0.56	0.36–0.86	0.008	0.48	0.31–0.74	0.001
LDH (IU/L)	1.84	1.00–3.38	0.050	—	—	0.195
B2MG (mg/dL)	1.08	1.03–1.14	0.002	1.07	1.03–1.10	<0.001
M-protein (g/dL)	—	—	0.475	—	—	0.802
Light chain disease	—	—	0.722	—	—	0.282
Nonsecretory type	—	—	0.504	—	—	0.247
Plasmacytoma	—	—	0.163	—	—	0.410
Cytogenetic risk (high)	—	—	0.134	—	—	0.083
Induction with novel agents*	—	—	0.542	—	—	0.711
ASCT	—	—	0.143	0.2	0.09–0.47	<0.001

*Induction with bortezomib, thalidomide, or renalidomide.

HR: hazard ratio; CI: confidence interval; ECOG: Eastern Cooperative Oncology Group; B2MG: *β*2-microglobulin; LDH: lactate dehydrogenase; ASCT: autologous stem cell transplantation; RDW: red blood cell distribution width.

**Table 3:** Multivariate analysis for progression-free survival.

	HR	95% CI	*P*
Age at diagnosis (year)	0.99	0.93–1.05	0.691
ECOG (≥2)	1.48	0.63–3.51	0.373
Cytogenetic risk (high)	**4.12**	**1.63–10.41**	**0.003**
B2MG (mg/L)	1.09	0.99–1.20	0.071
Albumin (<3.5 g/dL)	0.82	0.31–2.17	0.690
LDH (>normal)	1.35	0.56–3.26	0.499
Hemoglobin (>10 g/dL)	0.67	0.28–1.61	0.365
Calcium (>normal)	2.20	0.54–9.03	0.272
Induction with novel agents*	**0.34**	**0.14–0.81**	**0.014**
ASCT	0.96	0.28–3.25	0.945
High-RDW (>14.5%)	**3.21**	**1.24–8.32**	**0.016**

*Induction with bortezomib, thalidomide, or lenalidomide.

HR: hazard ratio; CI: confidence interval; ECOG: Eastern Cooperative Oncology Group; B2MG: *β*2-microglobulin; LDH: lactate dehydrogenase; ASCT: autologous stem cell transplantation; RDW: red blood cell distribution width.
